# Enhancing the Performance of Piezoelectric Wind Energy Harvester Using Curve‐Shaped Attachments on the Bluff Body

**DOI:** 10.1002/gch2.202100140

**Published:** 2022-10-28

**Authors:** Prakash Poudel, Saurav Sharma, M. N. M. Ansari, Rahul Vaish, Rajeev Kumar, Sobhy M. Ibrahim, P. Thomas, Chris Bowen

**Affiliations:** ^1^ School of Engineering Indian Institute of Technology Mandi Mandi Himachal Pradesh 175075 India; ^2^ Institute of Power Engineering Universiti Tenaga Nasional Kajang Selangor 43000 Malaysia; ^3^ Department of Biochemistry College of Science King Saud University Riyadh 11451 Saudi Arabia; ^4^ Central Power Research Institute Dielectric Materials Division Bangalore Karnataka 560080 India; ^5^ Department of Mechanical Engineering University of Bath Bath BA2 7AY UK

**Keywords:** curve‐shaped attachments, galloping, galloping instability, piezoelectric energy harvesting

## Abstract

This paper presents a piezoelectric wind energy harvester that operates by a galloping mechanism with different shaped attachments attached to a bluff body. A comparison is made between harvesters that consist of different shaped attachments on a bluff body; these include triangular, circular, square, Y‐shaped, and curve‐shaped attachments. Simulation of the pressure field and the velocity field variation around the different shaped bluff bodies is performed and it is found that a high pressure difference creates a high lift force on the bluff body with curve‐shaped attachments. A theoretical model based on a galloping mechanism is presented, which is verified by experiments. It is observed that the proposed harvester with curve‐shaped attachments provides the best performance, where the harvester with a curve‐shaped attachments provides the highest voltage and power output compared to the other shaped harvesters examined in this study. This paper provides a new concept for improving the power performance of the piezoelectric wind energy harvesters with modifications made on the bluff body.

## Graphical abstract

Different shaped attachments to the bluff body of the piezoelectric wind energy harvester operating on a galloping phenomenon is presented. The electrical voltage and power output can be enhanced with the different shaped attachments attached to the bluff body of the wind energy harvester.

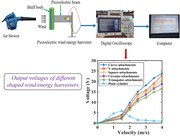

## 1 Introduction

Energy harvesting techniques on small scale are promising for the supply of continuous energy for wireless sensors, and low power portable electronic devices.^[^ ^1^, ^2^, ^3^, ^4^ ^]^ Powering these devices using traditional chemical batteries can have limitations as they have a limited lifespan and can have difficulty in replacement or battery management. Piezoelectric,^[^ ^5^ ^]^ triboelectric,^[^ ^6^ ^]^ and electromagnetic^[^ ^7^ ^]^ approaches to energy harvesting have been examined to provide the electrical energy for small electronic devices by utilizing the mechanical vibration of the system.

Flow induced energy harvesting has gained enormous popularity in micro‐electromechanical systems (MEMS) for a variety of industrial, biomedical and agricultural monitoring applications.^[^ ^8^, ^9^, ^10^ ^]^ Piezoelectric energy harvesters based on vibration due to wind energy is an important approach to energy harvesting because of its simple structure, without the need for any rotating components; it can also provide a high output in terms of voltage.^[^ ^11^, ^12^, ^13^ ^]^ Wind induced vibration involving the piezoelectric effect can be achieved by a variety of mechanisms, including vortex‐induced vibration (VIV),^[^ ^14^, ^15^, ^16^ ^]^ flutter,^[^ ^17^, ^18^, ^19^ ^]^ and galloping.^[^ ^20^, ^21^, ^22^ ^]^ Energy harvesters based on both vortex‐induced vibration as well as galloping phenomena have been studied in order to obtain optimum output power.^[^ ^23^, ^24^, ^25^ ^]^ Wind energy harvesters operating on vortex induced vibration and galloping generally involve the construction of a simple cantilever beam with piezoelectric sheets attached onto it. A bluff body is attached at the free end of the beam in order to produce vibration when wind flows over it. When the structures are subjected to wind flows, large amplitude oscillations at low frequency are produced. This phenomena known as galloping can lead to failures in structures with asymmetric cross sections. Failures of transmission lines in cold places, due to accumulation of snow on the wires, can lead to vibrations with high amplitude and is a good example of galloping.^[^ ^26^ ^]^ However, galloping in energy harvesting is considered to be beneficial as it produces large deflections of piezoelectric beams and subsequently higher electrical output.

Harvesting wind energy from a piezoelectric cantilever beam with a buff body attached to the free end is an effective approach for achieving galloping. A number of researchers have investigated the shape of the bluff body and proposed the optimum shapes to obtain a high output in simple wind harvesting systems. A comparison of equilateral triangular, square, rectangular and D‐shaped bluff bodies has been performed by Yang et al.^[^ ^27^ ^]^ A square shaped bluff body was found to perform the best for small scale galloping phenomenon, which was validated experimentally. Abdelkefi et al.^[^ ^28^ ^]^ examined the galloping on a cantilever beam with square, triangular and D‐shaped cross‐section. A distributed‐parameter model considering nonlinearities was used to study the effects of different shapes for optimum power at different electrical load resistances. A Y‐shaped bluff body made of thin sheets was designed and attached to the beam of a wind energy harvester by Liu et al.^[^ ^29^ ^]^ Several simulations and experiments were carried out by altering the angle of the blades, which confirmed that the proposed wind harvester performed better than the harvester with square cross‐section bluff body. Zhou et al.^[^ ^30^ ^]^ developed a bluff body with a curved shape thin plate to enhance the performance of a harvester at low cut‐in speed, and comparison were made with the square, triangle and D‐shaped harvester. The shape of the bluff body was modified to achieve an improved output of the wind energy harvester by attaching different shaped rods at certain angles in the bluff body. Hu et al.^[^ ^31^ ^]^ investigated the efficiency of the harvester by attaching two small rods to the main circular cylinder and the study showed that the output voltage can be greatly influenced by adding attachments to the bluff body. A high‐performance harvester with Y‐shaped attachments was proposed by Wang et al.^[^ ^32^ ^]^ and the transition of VIV to galloping phenomenon was studied. A comparison of the harvester with and without attachments revealed that a simple modification of the bluff body was sufficient to achieve optimum performance at low wind speeds. Wang et al.^[^ ^33^ ^]^ developed a harvester with spindle‐like and butterfly‐like cross‐sections for power enhancement at low speeds. Compared to other existing galloping harvesters, this proposed harvester was able to achieve high power by coupling both VIV and galloping phenomena.

Besides the high electrical voltage and power output, the reliability of the flow energy harvesters in the real applications is also essential. Gong et al.^[^ ^34^ ^]^ presented an energy harvester based on the vortex induced swing that is capable to measure the flow speed from the harvested power inside the water. Piezoelectric wind energy harvester that operates on the fluctuating wind speed was developed by Xu et al.,^[^ ^35^ ^]^ in where the wind was considered as a time dependent random process. The wind speed was studied using a mean component and a fluctuating component under stochastic averaging, and the framework developed in the study can be applied in real complex applications of highly efficient galloping energy harvesters. Zhang et al.^[^ ^36^ ^]^ developed an rotating piezoelectric energy harvester which is capable to power the sensor and also capable to detect faults on the rotating bearings.

In order to achieve the optimum performance, this paper designs a harvester with a bluff body that contains curve shaped attachments attached to it. A comparison of the harvester is undertaken with circular, triangular, square, and Y‐shaped attachments. A theoretical model of the harvester is developed, and a variety of experiments are carried out to validate the model, and undertake a parametric study of different design parameters. In Section 2, a mathematical model of the proposed wind energy harvester is discussed. The simulation of the harvester with different attachments is performed in Section 3. In Section 4, the experimental setup is described, along with the procedure involved. The experimental results obtained are then analyzed and compared among the harvesters with different shaped bluff bodies. We will see that the energy harvester with curved attachments in the bluff body has high performance with increased aerodynamic properties and high output voltage and power.

## 2 Mathematical Model

**Figure 1:**
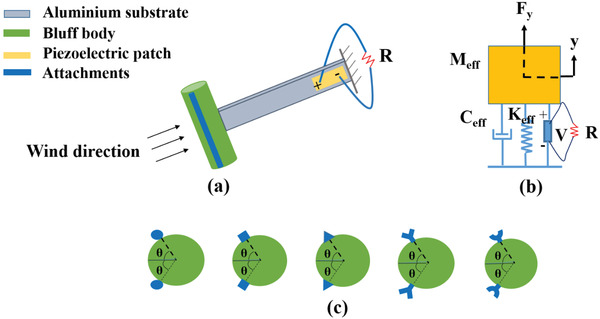
Schematic of the wind energy harvester: a) structural diagram with circular attachments to a bluff body. b) Equivalent lumped parameter model. c) Different shaped attachments (circular, square, triangular, *Y*‐shaped, and curve shaped).

The design of the harvester is based on galloping phenomena that occurs when air flows through the bluff body of the harvester. **Figure** **1**a,b shows a piezoelectric wind energy harvester that consists of a piezoelectric beam fixed at one end, and a bluff body attached at the other end. The direction of the wind is perpendicular to the surface of the bluff body, as shown in Figure 1. Encouraged by the design of bluff body with two circular rods at different angles,^[^ ^37^ ^]^ we present the concept of adding different shaped attachments to the main circular bluff body for obtaining efficient galloping, and hence the improved electrical output. Different shaped attachments, namely circular, square, triangular, Y‐shaped, and curved‐shaped are used in this study, and are shown in Figure 1c. Considering Euler–Bernoulli beam theory, the piezoelectric effect, Kirchhoff's law and self‐induced galloping vibration, a distributed parameter model of the harvester can be obtained which can be further converted into lumped parameter model.^[^ ^12^, ^32^ ^]^ The modeling of piezoelectric wind energy harvester involves the piezoelectric effect with fluid flow interaction between describing fluid dynamics and structural mechanics. Therefore, it is suitable to model the system as a lumped parameter, Figure 1b, which greatly simplifies the complexity of the system.

The structure is modified into a single degree of freedom (sdof) model with equivalent mass, stiffness and damping constants as shown in Figure 1b. The system can be described by a set of coupled electromechanical equations, and can be written as

(1) $$\begin{array}{c} M_{\text{eff}}\ddot{y}\left(t\right)+C_{\text{eff}}\dot{y}\left(t\right)+K_{\text{eff}}y\left(t\right)+\theta_{c}V\left(t\right)\;=F_{y}\left(t\right) \end{array}$$

(2) $$\begin{array}{c} C_{p}\dot{V}(t)+\frac{1}{R}V(t)-\theta_{c}\dot{y}(t)=0 \end{array}$$

where the equivalent mass, $M_{\text{eff}}=\left(\frac{33}{140}\right)\;m_{1}+m_{2}+m_{3}$ with *m*_1_, *m*_2_, and *m*_3_ are the masses representing the piezoelectric beam, bluff body and the two attachments fixed to the bluff body, respectively. The attachments are fixed on the bluff body and make an angle of 2θ = 120° with each other, as shown in Figure 1c. The equivalent damping *C*_eff_ = 2*ξw_n_M*_eff_ and equivalent stiffness $K_{\text{eff}}=w_{n}^{2}\;M_{\text{eff}}$ are determined through experiments. A logarithmic decrement technique is employed to obtain the damping ratio, ξ. Similarly, a free decay test is used to calculate the natural frequency, *w_n_* of the system. Here, *y*(*t*) is the linear displacement of the bluff body in the direction perpendicular to that of wind flow. The output parameter of the harvester is the voltage *V*(*t*), which is measured across the load resistance, *R*. Using the open circuit condition, $I\;=\frac{V}{R}\;=\;0$ and the short circuit criteria, *V* = 0 and replacing the respective values in Equations (1) and (2), we can obtain the electromechanical coupling coefficient as

(3) $$\begin{array}{c} \theta_{c}=\sqrt{\left(w_{\text{oc}}^{2}-w_{\text{sc}}^{2}\right)M_{\text{eff}}C_{p}} \end{array}$$

The natural frequency, *w*_oc_ defined at the open circuit condition and the natural frequency, *w*_sc_ defined at the short circuit condition can be calculated by experimental measurements. The capacitance of the piezoelectric patch, *C*_p_ is obtained from the manufacturer's formula.

By performing experiments and using the principles discussed above, the model parameters of the energy harvester are obtained. The mass of a cantilever beam and a bluff body was measured to be 2.54 and 2.52 g, respectively. Similarly the mass of the curve‐shaped attachments was 3.45 g. The effective mass, effective damping, and the effective stiffness of the lumped parameter model are 7.5 g, 0.0059 N (m^−1^ s^−1^), and 6.8359 N m^−1^, respectively. In addition, the electromechanical coupling coefficient, θ_c_ is calculated to be 2.24 × 10^–5^ N V^−1^. The capacitance, *C*_p_ of a PZT piezoelectric patch is 1.3574 × 10^–8^ F.^[^ ^38^ ^]^ In addition, the natural frequency, *f*_oc_ measured at open circuit condition and the natural frequency, *f*_sc_ measured at short circuit criteria of the system are calculated as 4.821 and 4.814 Hz, respectively. The damping ratio, ξ is experimentally measured to be 0.013.

**Figure 2:**
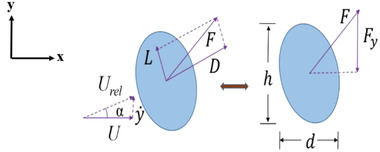
A bluff body subjected to galloping.

The aerodynamic force, *F_y_* (*t*) that acts on the bluff body due to galloping phenomenon is given as^[^ ^39^, ^40^ ^]^

(4) $$\begin{array}{c} F_{y}\left(t\right)\;=\frac{1}{2}\;\rho U^{2}dhC_{F}{\;}_{y} \end{array}$$

where *d* and *h* are the frontal dimensions of the bluff body facing the direction of wind, ρ is the density of the air and *U* represents the speed of the wind. *C_F_*_*y*_ denotes the coefficient of the aerodynamic force in *y*‐direction. The aerodynamic force coefficient is an important parameter in design of the energy harvester and depends on the shape of a bluff body. The galloping phenomenon that is responsible for the motion of the bluff body can be explained by considering the force acting on a body, other than circular cross‐section as shown in **Figure** **2**.

The force *F*_*y*_ acting on a bluff body is expressed in terms of lift and drag forces^[^ ^39^ ^]^ as

(5) $$\begin{array}{c} F_{y}=\;-\left(L\cos\alpha +D\sin\alpha \right) \end{array}$$

Assuming a quasi‐static hypothesis, the aerodynamic force acting on the oscillating body is equivalent to the force acting on a steady body, measured at an equivalent angle of attack, considering low oscillation of the body. For a body undergoing only translational vibration motion, without any rotational motion, the angle of attack α is given as

(6) $$\begin{array}{c} \alpha \;=\tan^{-1}\left(\frac{\dot{y}\left(t\right)}{U}\right) \end{array}$$

The value of *C_F_*_*y*_ depends on the angle of attack and an approach to obtain its value is to express it in a cubic polynomial form as^[^ ^39^, ^41^ ^]^

(7) $$\begin{array}{c} C_{F_{y}}=\;\left(a_{1}\frac{\dot{y}\left(t\right)}{U}+a_{3}{\left(\frac{\dot{y}\left(t\right)}{U}\right)}^{3}\right) \end{array}$$

The empirical coefficients *a*_1_ and *a*_3_ are achieved by curve fitting of $C_{F_{y}}$ versus α curve. The plot of $C_{F_{y}}$ versus α curve is obtained experimentally in a static test with varying angle of attack. Den Hartog^[^ ^42^ ^]^ explained the instability of the galloping phenomenon, which can be expressed as

(8) $$\begin{array}{c} \frac{\partial C_{L}}{\partial \alpha }+C_{D}<0 \end{array}$$

where *C_L_* = 2*L*/(*ρU* ^2^ *hd*) is the lift coefficient, *C*_D_ = 2D/(*ρU* ^2^ *hd*) is the drag coefficient. L and D are the lift and drag forces acting on a body respectively. Equation (8) indicates that for a specified orientation of a bluff body with a small oscillation and a small change in angle of attack, the galloping instability of the body requires negative slope of the lift coefficient.^[^ ^43^ ^]^

If we consider the rotation effect of the bluff body, the polynomial expansion of $C_{F_{y}}$ can be revised as

(9) $$\begin{array}{c} C_{F_{y}}=\;\left(a_{1}\left(\frac{\dot{y}\left(t\right)}{U}+y^{\prime }\left(t\right)\right)+a_{3}{\left(\left(\frac{\dot{y}\left(t\right)}{U}\right)+y^{\prime }\left(t\right)\right)}^{3}\right) \end{array}$$

where *y*′ (*t*) = *μy*(*t*) and, μ is the coefficient that relates the transverse displacement and rotation at the free end of the cantilever beam and is given as, μ = 1.5/*l*.

A linear analysis on the coupled electromechanical equations is carried out to obtain the solution for the energy harvester model. For this, we define a state vector X as

(10) $$\begin{array}{c} X\;=\left\{\begin{array}{c} x_{1}\\ x_{2}\\ x_{3} \end{array}\right\}\;=\left\{\begin{array}{c} y\left(t\right)\\ \dot{y}\left(t\right)\\ V\left(t\right) \end{array}\right\} \end{array}$$

Rearranging Equations (1) and (2), we can obtain the governing equations harvester in matrix form as

(11) $$\dot{X}=\left\{\begin{array}{c} \dot{y}\left(t\right)\\ -\frac{C_{\text{eff}}}{M_{\text{eff}}}\dot{y}\left(t\right)\\ \frac{\theta_{c}}{C_{p}}\dot{y}\left(t\right)-\frac{V\left(t\right)}{RC_{p}} \end{array}-\frac{K_{\text{eff}}}{M_{\text{eff}}}y\left(t\right)-\frac{\theta_{c}}{M_{\text{eff}}}V\left(t\right)+\frac{\rho U^{2}dh}{2M_{\text{eff}}}\left(a_{1}\left(\frac{\dot{y}\left(t\right)}{U}+µy\left(t\right)\right)+a_{3}{\left(\left(\frac{\dot{y}\left(t\right)}{U}\right)+µy\left(t\right)\right)}^{3}\right)\right\}$$

Equation (11) is solved using MATLAB Simulink for the vibration response of the beam along with calculation of output voltage across different electrical load resistances. The average power output of the harvester is calculated using, $=\frac{V_{\text{rms}}^{2}}{R}$.

The theoretical model discussed above incorporates the both electromechanical and the aerodynamic behavior of the piezoelectric wind energy harvester system. The electromechanical model presents the coupled equations, based on lumped parameter model and predicts the electrical voltage and electrical power of the energy harvester. The force that drives the wind energy harvester is defined based on the aerodynamic model of the wind energy harvester. The force acting on the wind energy harvester with different shaped bluff bodies is different, depending on the values of the aerodynamic force coefficient, *C_F_*_*y*_. The empirical coefficients, *a*_1_ and *a*_3_ related to the aerodynamic force coefficients are calculated experimentally and listed on **Table** **1**. The complete theoretical model presented here predicts the mechanism of piezoelectric wind energy harvester under galloping phenomenon.

**Table 1:** Empirical coefficients *a*_1_ and *a*_3_ for different shaped bluff bodies

Bluff body shapes	Plain circular	Triangular attachments	Circular attachments	Square attachments	*Y*‐attachments	Curve attachments
*a*_1_	2.9	0.9	2.09	1.5	2.17	2.29
*a*_3_	−178	−1628	−1268	−1552	−913	−726

## 3 Simulation Analysis of the Bluff Body

To demonstrate the mechanism of galloping based wind energy harvesting using different shaped attachments, a two dimensional model was developed. We performed simulations with the standard k‐ε turbulence model in order to obtain better computational accuracy with high stability. The length and width of the computational domain used for the simulation are 100 mm and 80 mm respectively. A free triangular mesh is adopted, with three different meshing sizes, namely 65 652 (coarse), 89 532 (medium), and 102 568 (fine). When the coarse mesh is replaced by a medium mesh, the lift and drag coefficients changes by nearly 12%. Similarly, when the resolution of the medium mesh is adjusted to fine, the coefficients of lift and drag forces is changed by less than 2%. Thus, the medium mesh resolution is chosen for our simulation analysis.

**Figure 3:**
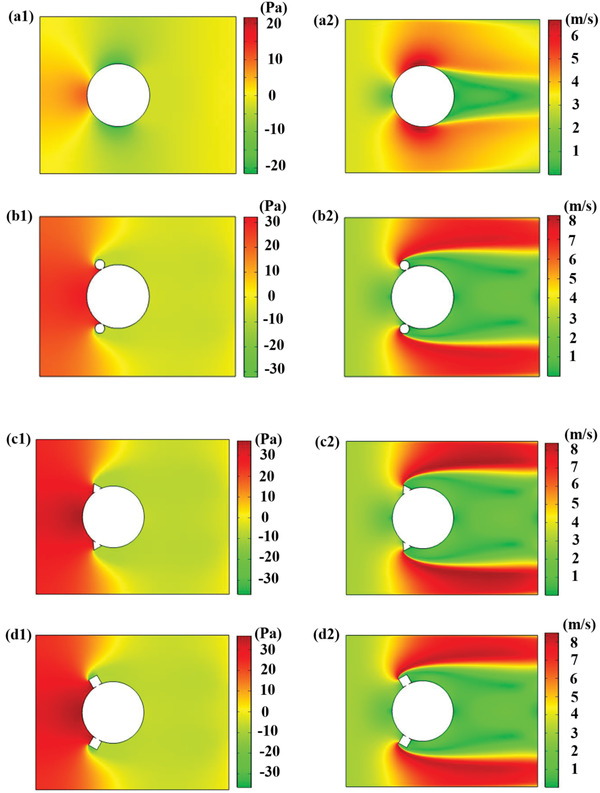
Continued.

The incoming air velocity at the inlet boundary is taken as 3 m s^−1^ and the air is supposed to flow in the direction perpendicular to the inlet domain. The pressure is considered zero at the outlet boundary of the domain and the top and bottom boundaries are considered to be fixed. The variation of the pressure field and the velocity field around the bluff body with different shaped attachments is shown in **Figure** **3**. Figure 3a shows the pressure and velocity variation when the air blows past the plain cylindrical bluff body. The pressure difference between the upstream and the downstream side of the bluff body reveals that there exists a lift force on the bluff body, which creates the transverse force component that is necessary for the galloping mechanism. Recently, Liu et al.^[^ ^44^ ^]^ performed a CFD analysis based on COMSOL on a circular bluff body with double flat plates placed ahead of the bluff body for utilizing the wake flow produced by such plates for improved harvesting performance. Figure 3b1–f1 shows the pressure field variation around the bluff body with different shaped attachments, and the velocity field variation can be observed in Figure 3b2–f2. If we observe the pressure variation in bluff bodies with different shaped attachments, it is found that the minimum pressure occurs at the downstream of the body. The occurrence of negative pressure and high lift force will make the system aerodynamically unstable, and this instability will eventually increase the amplitude of vibration of the body. The variation of velocity with maximum value at the upper and lower sides of the body, whereas the minimum value just behind the bluff body that is observed for different shaped attachments signifies that there is vibration in the bluff body.

## 4 Experimental Studies

**Figure 4:**
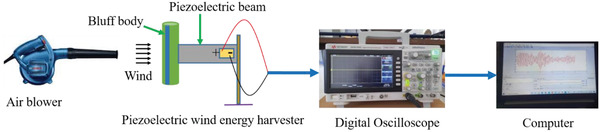
Experimental setup of piezoelectric wind energy harvester.

In order to verify the results achieved from the theoretical model, an experimental setup was developed, and a range of experiments were performed in an open atmosphere. **Figure** **4** shows the experimental setup used for energy harvesting using different shaped attachments on the bluff bodies. A centrifugal air blower was used to supply the wind required to drive the harvester. The speed of the wind was measured using digital anemometer. The required speed of wind is maintained by making appropriate adjustment of the blower. A PZT‐5A (SP‐5A, India) lead zirconate titanate piezoelectric sheet with dimensions of 50 × 20 × 0.4 mm^3^ was placed at one end of pure aluminium beam with dimensions of 200 × 25× 0.6 mm^3^; this a is relatively soft ferroelectric materials that has high piezoelectric activity, making it suitable for sensing and harvesting applications. A circular bluff body with a height of 120 mm and a diameter of 32 mm was made of expanded polystyrene (EPS) material with low density and the different shaped attachments required were fabricated using a 3D printer. The material used in the attachments is polylactic acid (PLA) and the length was made equal to that of the bluff body with diameter 5 mm for circular attachments. Similarly, the sides of triangular and square attachments were chosen to be 5 mm and similar dimensions was considered for Y‐shaped and curve‐shaped attachments. The output voltage obtained across the electric load resistance was measured using a digital oscilloscope (InfiniiVision DSO‐X 3034A) with an input impendence of 10 MΩ.

**Figure 5:**
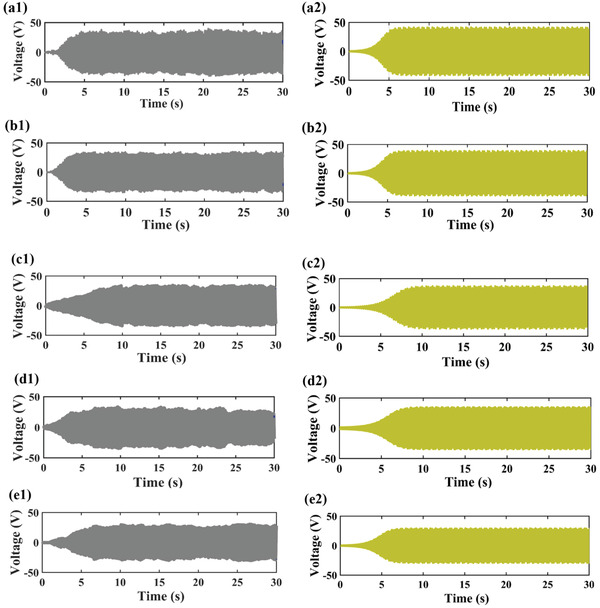
Experimental and simulation output voltage at 4 m s^−1^ wind speed: a1–e1) Experiment; a2–e2) Simulation of different shaped harvester; a) curved attachments; b) Y‐shaped attachments; c) square attachments; d) circular attachments; e) triangular attachments.

The experimental results obtained are analyzed and compared with the simulation results. The experimental output voltage of the harvester with curved shaped attachments is found to be ≈25 V and the simulated output voltage is about 29 V when the speed of wind is kept at 4 m s^−1^, as shown in **Figure** **5**a. The difference in the simulated and experimental value can be due to the assumptions used in modeling of the harvester. The modeling is based on the lumped parameter model and a quasi‐steady hypothesis is assumed in the derivation of transverse force component with small angle of attack. In addition, the experiments are performed in an open environment, where it is difficult to predict the wind behavior accurately. Figure 5b compares the experimental and simulated output voltage of the harvester with Y‐shaped attachments. Similarly, Figure 5c–e illustrate the comparisons for square, circular and triangular attachments respectively. If we compare the output voltage produced by different shaped harvesters, we can conclude that the harvester with curved shaped attachments provides the best performance, while the harvester with triangular attachments leads to the lowest output voltage and power. It can be seen that it requires a few seconds for the harvesters to produce stable output voltage for both simulation and experiment, as seen in Figure 5.

**Figure 6:**
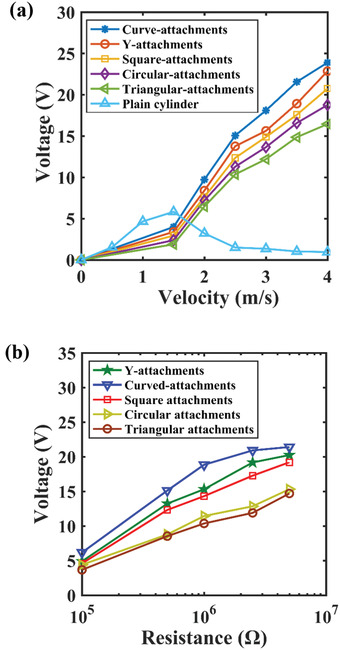
Experimental comparison of the output voltage with different attachments with: a) wind velocity; b) load resistance in log scale at 4 m s^–1^ wind speed.

**Figure** **6**a illustrates the variation of output voltage with wind speed produced by the harvesters with different attachments. It can be seen that the output voltage is increased when the speed of wind is increased for the harvesters with attachments subject to galloping phenomenon. Galloping occurs when the wind speed is greater than the threshold value required for galloping and the experimentally measured threshold value is ≈1.65 m s^−1^. However, for the harvester without any attachments, there is vortex‐induced vibration (VIV) phenomena. The maximum output voltage for the harvester with a plain circular bluff body occurs at a velocity where a lock‐in region exists. In this lock‐in region, the oscillating frequency is locked to the natural frequency of the harvester. Figure 6a demonstrates that the lock‐in region exists at ≈1.2–1.5 m s^−1^, where the harvester has maximum voltage of 6 V. It can also be observed that a post‐synchronization stage exists after the lock‐in region, where the output voltage starts decreasing with increasing velocity. It is important to understand that the harvester operating under vortex‐induced vibration will enter the lock‐in region at a velocity less than that required for galloping of the harvesters with different shaped harvesters. However, the harvesters operating under galloping will perform at a higher velocity as compared to the harvester with only a plain circular harvester. The variation of output voltage with different load resistance is shown in Figure 6b. Experiments were carried out for five different load resistances (0.1, 0.5, 1, 2.5, and 5 MΩ) and the results show that the output voltage increases when we shift from 0.1 to 1 MΩ resistance, but it remains almost constant while resistance is increased from 1 to 5 MΩ. The output voltage of the harvester with curve‐shaped attachments provides highest output voltage with different load resistances.

**Figure 7:**
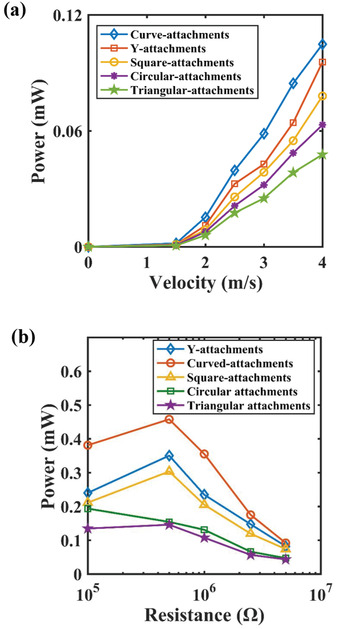
Experimental comparison of the power output of the harvester with different attachments with: a) wind velocity at 5 MΩ resistance; b) load resistance in log scale at 4 m s^–1^ wind speed.

The variation of the power output with wind speed and load resistance is illustrated in **Figure** **7**a,b respectively. The power output provided by the harvesters operating at wind speeds lower than 1.5 m s^–1^ is low and beyond it, the power increases with increasing the wind speed. The power output of the harvester with curve‐shaped attachments provides the maximum value of ≈0.105 mW while operating at a wind speed of 4 m s^–1^. Similarly, a lower power output is generated by harvester with triangular attachments and its value is 0.048 mW. If we observe the variation of power output with electric load resistance, it is found that the harvester with curve‐shaped attachments performs best under different load resistances. The maximum power output is obtained with curve‐shaped attachments operating at 4 m s^–1^ wind speed with an electrical load resistance of 0.5 MΩ and its value is 0.46 mW as seen in Figure 7b.

**Figure 8:**
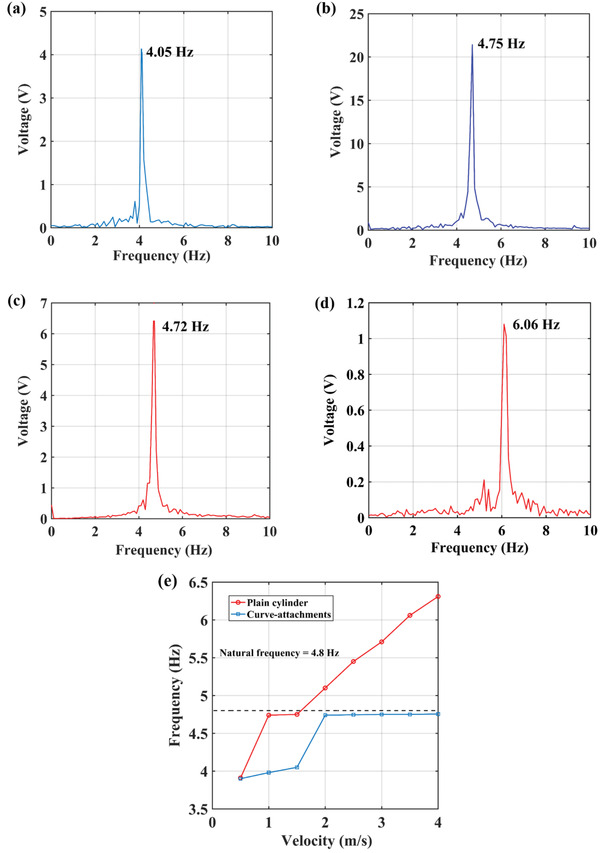
Frequency domain diagrams: a) plain cylinder at 1.5 m s^–1^; b) plain cylinder at 3.5 m s^–1^; c) curve‐attachments at 1.5 m s^–1^; d) curve‐attachments at 3.5 m s^–1^; e) comparison of frequencies of plain cylinder and curve‐attachments at different wind speeds.

The frequency domain diagram is plotted in **Figure** **8**. The frequencies of vibration of the wind energy harvesters at different velocities are obtained using the fast Fourier transform (FFT) method. Figure 8a,b shows the frequency of vibration of the harvester with a curve‐shaped attachments, at the wind speeds of 1.5 and 3.5 m s^–1^, and are found to be 4.05 and 4.75 Hz, respectively. Similarly, the frequency of vibration of the harvester with a cylindrical shaped bluff body, at the wind speeds of 1.5 and 3.5 m s^–1^, and are found to be 4.72 and 6.06 Hz, respectively, as shown in Figure 8c,d. The plot of frequencies of the two different harvesters consisting a plain cylindrical shaped bluff body and curve‐shaped attachments to the bluff body, at different wind speeds is shown in Figure 8e. The natural frequency of vibration for both shaped harvesters is 4.8 Hz. It can be seen that for the harvester with curve‐shaped attachments, the frequency of vibration is less than the natural frequency of the system at low wind speeds. As the wind speed increases beyond 2 m s^–1^, the frequency of vibration is close to the natural frequency of the harvester, thus providing the oscillations with higher amplitude. In case of the harvester without attachments, the frequency of vibration is close to the natural frequency at the wind speed of range 1 to 1.5 m s^–1^. The frequency of the harvester is locked to the natural frequency of the system in this wind speed range, thus providing large voltage output from large amplitude oscillations. As the wind speed is increased beyond 1.5 m s^–1^, the oscillating frequency of the harvester also increases, as shown in Figure 8e.

**Figure 9:**
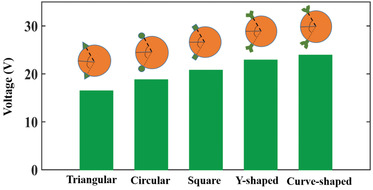
Maximum output voltage of harvesters with different shaped attachments measured at 4 m s^–1^ wind speed.

**Figure** **9** represents the bar diagram showing the comparison of the output voltage produced by the harvesters with different shaped attachments under study. The output voltage and the output power of the piezoelectric wind energy harvester depends on the amplitude of oscillation of the bluff body attached to the beam. The shape of the bluff body greatly influences the oscillations produced because the force acting on the bluff body depends on it. In case of curve‐shaped attachments, the force coefficient acting on the bluff body is higher because the lift force that drives the harvester is more in compared to other shaped harvester. It can be observed that the curve‐shaped attachments provides a high output voltage and power whereas, the triangular attachments provides a comparatively low output voltage and power.

## 5 Conclusions

Wind energy harvesting approaches based on aerodynamic instabilities are widely used for providing power to wireless sensor networks and small electronic devices. Research has revealed that the galloping based harvesters are favorable for harvesting ambient wind energy due to its simple structure and ease of device fabrication. In this paper, a comparative analysis of different shaped attachments on a bluff body is performed to illustrate their effect on the output voltage and output power of a piezoelectric wind energy harvester. A theoretical model considering lumped parameters is developed, which verified with results obtained from experiments, with good agreement. The voltage and power produced by the harvester with several different shaped attachments is compared; these include circular, triangular, square and *Y*‐shaped attachments. The harvester with curved‐shaped attachments provides an output voltage of 25 V and a power output of 0.105 mW when operating at a wind speed of 4 m/s, which is higher than the output produced by harvesters with other shaped attachments under consideration. Similarly, the overall output produced by the harvester consisting of triangular attachments on a bluff body is the lowest, when compared with other shaped attachments. Simulation analysis performed on the bluff bodies with different attachments leads to a high pressure difference on a bluff body with curved attachments compared to other attachments producing more vibration required for improved performance. The proposed curved attachments on a bluff body lead to improved aerodynamic efficiency with design flexibility. We have performed the experimental analysis in an open environment which allows good potential for different applications of harvested power as it is not always possible to harvest wind power in a wind tunnel.

## Conflict of Interest

The authors declare no conflict of interest.

## Data Availability

The data that support the findings of this study are available on request from the corresponding author. The data are not publicly available due to privacy or ethical restrictions.
